# Supplementary figures and images for: A decade of stability for wMel Wolbachia in natural Aedes aegypti populations
Source: PLoS Pathog. 2022 Feb 23;18(2):e1010256. doi: 10.1371/journal.ppat.1010256 (PMC8901071; doi:10.1371/journal.ppat.1010256)

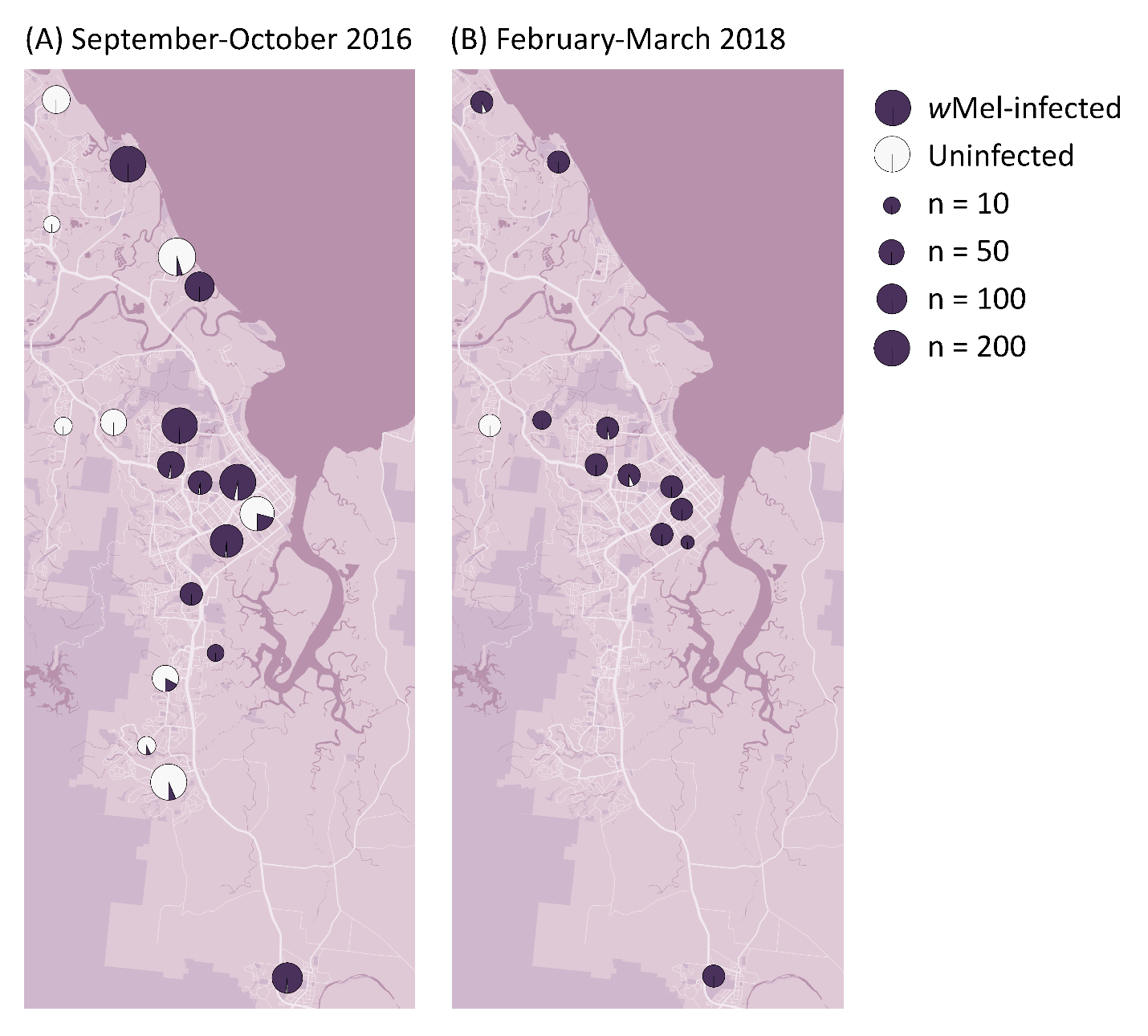

Supplement: S1 Fig — wMel Wolbachia infection frequencies in Cairns in (A) 2016 and (B) 2018 sampled through ovitrapping. Contains information from OpenStreetMap and OpenStreetMap Foundation, which is made available under the Open Database License. (TIF) [file ppat.1010256.s001.tif]
