# Supplementary material for: A decade of stability for wMel Wolbachia in natural Aedes aegypti populations
Source: PLoS Pathog. 2022 Feb 23;18(2):e1010256. doi: 10.1371/journal.ppat.1010256 (PMC8901071; doi:10.1371/journal.ppat.1010256)
Supplement: Attachment — Submitted filename: Reviewer comments.docx [file ppat.1010256.s004.docx]

Response to reviewers

Reviewer comments **(responses in bold)**

Reviewer #1: Although the manuscript provide new and relevant information, some additional details are required in specific sections of the manuscript.

Line 77. Authors mention that in some locations, Wolbachia infection has remained at an intermediate frequency or dropped out, requiring supplemental releases. For sure, environmental, entomological and likely human behavior aspects influence this outcome. I'd recommend authors to add a few sentences in the Discussion section correlating how their lab-based results could help explaining (at least partially) the reason why some areas would still need supplemental releases to achieve a stable invasion.

**As requested, we have added a few lines to the end of the discussion (lines 314-321) explaining that variation in mosquito genetics may influence the phenotypic effects of wMel, and this could help to explain variable establishment success. We have distinguished background effects in response to Wolbachia and indirect effects stemming from host genetic material introduced during releases now (lines 327-331). Unfortunately, the results of this study alone are not sufficient given that our data are restricted to mosquitoes collected from a single region, but we have added some speculation about other releases.**

Line 178: In the phenotypic comparison assays, authors observed fecundity and egg hatch proportions during four consecutive gonotrophic cycle and compared data using GLMs. One of the most important assumptions of Generalized linear models is the independence among data points. From a personal perspective, I wouldn't say the number of eggs from a given gonotrophic cycle is fully independent from the previous one, especially when mosquito females are kept in cages.

**We agree in this case that the data were not fully independent and we have therefore now performed separate analyses for each gonotrophic cycle (see lines 174-178). We find effects of population, but only in one out of the four gonotrophic cycles for each trait, and this is now noted.**

Line 356: On this manuscript, authors report the spatiotemporal variation of Wolbachia density in 4th instar larvae hatched from ovitraps collected across Cairns suburbs in February-March 2018. However, the research group leading this manuscript has data from older field samplings. Would still be possible to evaluate potential changes/fluctuations of Wolbachia density over this 10 years period? Or considering data already published, the use of different primers make a direct comparison not recommended?

**Unfortunately we no longer have access to these historical samples in order to perform a direct comparison of density. For accurate comparisons of density, samples need to be run on the same plate (or spread evenly across multiple plates) and with the same batch of PCR master mix and primers. These older samples would have been run with small differences in reagents and PCR conditions and run by different people, so we do not believe that it is valid to compare them directly.**

Line 398: What does it mean? Adding food ad libitum would make the water cloudy, strongly affecting rearing conditions and thus larval mortality.

**We now specify (line 415-416) that trays were checked daily, with excess food removed to prevent overfeeding. We did not observe cloudy water in any tray during the experiments, with little larval mortality.**

Line 421: Authors claim they allowed multiple generations of recovery from tetracycline treatment before start the experiments. But I understood it was for only two generations.

**We now specify that we allowed at least two generations of recovery.**

Reviewer #2: I think the ‘Discussion’ could use an additional speculative section on why other places ‘don’t look like Cairns’. Why if this symbiont has so little effect on populations is it falling out of Vietnamese and Brazilian populations? What is known about fitness in these other regions?

**Please see our response above to reviewer 1; we have added a few lines to the end of the discussion (lines 314-321) explaining that variation in mosquito genetics may influence the phenotypic effects of wMel, and this could help to explain variable establishment success. We also now cite two papers which look at fitness effects of wMel in other mosquito backgrounds (Brazil and New Caledonia) and we emphasize the unusual situation in North Queensland where resistance to pesticides has not developed whereas this factor is important in other countries including those where invasion has not been straightforward (lines 327-331). Note that while Brazil and New Caledonia studies are broadly consistent with data from Australian mosquitoes, there haven’t been direct comparisons across multiple backgrounds.**
